# Evolution of GHF5 endoglucanase gene structure in plant-parasitic nematodes: no evidence for an early domain shuffling event

**DOI:** 10.1186/1471-2148-8-305

**Published:** 2008-11-03

**Authors:** Tina Kyndt, Annelies Haegeman, Godelieve Gheysen

**Affiliations:** 1 Department of Molecular Biotechnology, Ghent University, Coupure links 653, 9000 Ghent, Belgium

## Abstract

**Background:**

Endo-1,4-beta-glucanases or cellulases from the glycosyl hydrolase family 5 (GHF5) have been found in numerous bacteria and fungi, and recently also in higher eukaryotes, particularly in plant-parasitic nematodes (PPN). The origin of these genes has been attributed to horizontal gene transfer from bacteria, although there still is a lot of uncertainty about the origin and structure of the ancestral GHF5 PPN endoglucanase. It is not clear whether this ancestral endoglucanase consisted of the whole gene cassette, containing a catalytic domain and a carbohydrate-binding module (CBM, type 2 in PPN and bacteria) or only of the catalytic domain while the CBM2 was retrieved by domain shuffling later in evolution. Previous studies on the evolution of these genes have focused primarily on data of sedentary nematodes, while in this study, extra data from migratory nematodes were included.

**Results:**

Two new endoglucanases from the migratory nematodes *Pratylenchus coffeae *and *Ditylenchus africanus *were included in this study. The latter one is the first gene isolated from a PPN of a different superfamily (Sphaerularioidea); all previously known nematode endoglucanases belong to the superfamily Tylenchoidea (order Rhabditida). Phylogenetic analyses were conducted with the PPN GHF5 endoglucanases and homologous endoglucanases from bacterial and other eukaryotic lineages such as beetles, fungi and plants. No statistical incongruence between the phylogenetic trees deduced from the catalytic domain and the CBM2 was found, which could suggest that both domains have evolved together. Furthermore, based on gene structure data, we inferred a model for the evolution of the GHF5 endoglucanase gene structure in plant-parasitic nematodes. Our data confirm a close relationship between *Pratylenchus *spp. and the root knot nematodes, while some *Radopholus similis *endoglucanases are more similar to cyst nematode genes.

**Conclusion:**

We conclude that the ancestral PPN GHF5 endoglucanase gene most probably consisted of the whole gene cassette, i.e. the GHF5 catalytic domain and the CBM2, rather than that it evolved by domain shuffling. Our evolutionary model for the gene structure in PPN GHF5 endoglucanases implies the occurrence of an early duplication event, and more recent gene duplications at genus or species level.

## Background

Endo-1,4-beta-glucanases (EC 3.2.1.4), or endoglucanases, can degrade the beta-1,4-linkages of cellulose, the most abundant component of plant cell walls. The endoglucanases are classified in different glycosyl hydrolase families (GHF) on the basis of sequence similarity and hydrophobic cluster analysis [1]. Animal endoglucanases belong to three structurally and presumably phylogenetically unrelated families: GHF5, GHF9 and GHF45 [2]. GHF5 genes are found in various plant-parasitic bacteria and nematodes [3-6] from the order Rhabditida (infra-order Tylenchomorpha), which is part of one of the three evolutionary independent plant-parasitic nematode clades [7]. In the other two clades (Dorylaimida and Triplonchida), no GHF5 endoglucanases have been found so far. Next to plant-parasitic nematodes (PPN), some protists, plants and fungi, as well as two beetle species [8,9] have GHF5 endoglucanases. The domain structure of GHF5 PPN endoglucanases shows remarkable similarity to particular bacterial genes that consist of a GHF5 catalytic domain, a linker and a carbohydrate-binding module of family 2 (CBM2) [10,11]. Interestingly, some GHF5 PPN endoglucanases lack the CBM. Other bacterial endoglucanases as well as those from plants and fungi contain different types of carbohydrate-binding modules, while currently known GHF5 endoglucanases of protists and beetles comprise no CBM.

Horizontal gene transfer (HGT) from bacteria has been proposed to explain the origin of GHF5 endoglucanases in nematodes [12]. However, although HGT was also suggested to lie at the origin of GHF9 cellulases, this hypothesis was rejected based on recent evidence showing that there was a GHF9 cellulase gene in the last common ancestor of bilaterian animals [2]. At present, no hard evidence for the HGT hypothesis of GHF5 endoglucanases has been found. An evolutionary scheme proposed for GHF5 genes of sedentary nematodes by Ledger *et al. *[13] hypothesized that after the possible HGT event, the endoglucanase genes must have been duplicated several times, and in some cases a sequential loss of the linker and CBM occurred. This model assumes that the domain structure of the ancestral PPN GHF5 endoglucanase consisted of a catalytic domain, a linker and a CBM, although the structure of this ancestral gene has never been firmly established. A possible HGT event from bacteria could have involved the whole cassette as well as only the CBM2. The fact that the GHF5 domain has also been found in other eukaryotic lineages can be seen as evidence for its presence in the ancestral eukaryote, and a subsequent combination with a horizontally-acquired CBM2 through 'early' (i.e. in one of the ancestral nematodes) domain shuffling could have resulted in the presence of proteins with both domains in PPN. For bacterial genes, domain shuffling between GHF5 and CBM2 domains has already been proven [14].

Domain shuffling [15] is a special form of exon shuffling, in which new combinations of exons (encompassing a complete protein domain in the case of 'domain shuffling') from unrelated genes can be created by recombination within the intervening intron sequences, yielding rearranged genes with altered function [16]. Since the discovery of introns there has been considerable debate concerning their origin and possible involvement in exon/domain shuffling events.

The original introns-early concept states that (nearly) all introns have been inherited by eukaryotic genes from prokaryotic ancestors [16-19]. The difference in exon/intron-structure among homologous genes is then considered to be mostly due to differential intron loss. Observed relationships between intron locations and protein structures (module boundaries) are used as evidence for the ancient origin of introns and for their role in exon/domain shuffling [20].

Cavalier-Smith strongly disagrees with the introns-early view [21]. On the basis of phylogenetic considerations and the unique requirements of the eukaryotic nucleus, he suggests that the original genes were uninterrupted. He and other proponents of the introns-late theory argue that the introns observed in extant genes occur at far too many positions to have been present all together in a common ancestral gene [22]. In addition, they argue that the introns-early idea is hard to reconcile with the absence of spliceosomal introns and the spliceosome itself in prokaryotes (explained by 'introns-early' debaters as the result of genome streamlining in prokaryotes). The introns-late concept states that introns are an invention of eukaryotes and new introns have been continuously emerging during eukaryotic evolution [23]. This theory is supported by the discovery of a conserved sequence at exon-intron boundaries, called the protosplice-site (MAG/R) [24], which is believed to be a specific target site for newly gained introns, or a preferential site for intron fixation.

A synthetic theory of intron evolution has been put forward by de Souza [25]. This intermediate position proposes that a subset of present-day introns is ancient, but in addition, allows the emergence of some new introns. The so-called 'many-introns-early-in-evolution' theory places the origin of numerous spliceosomal introns during (and maybe playing a role in) eukaryotic radiation [26]. Recent studies provide high support for this theory [27-29]. The lack of conserved intron positions in ancient paralogues was suggested to demonstrate that the invasion of group II self-splicing elements (which were to become spliceosomal introns in the future) occurred during gene duplication, most probably in the earliest phase of eukaryotic evolution [30].

In order to determine the domain structure of the ancestral PPN GHF5 endoglucanase gene present in the PPN of the phylum Tylenchomorpha, the evolution of the GHF5 catalytic domain and the CBM2 cassette was investigated through phylogenetic analyses including sequences from fungi, plants, protista and bacteria.

To study the subsequent evolution of the GHF5 endoglucanase gene family within nematodes, intron positions were used as phylogenetic characters and a model was developed for their evolution.

Until now, evolutionary models for PPN GHF5 endoglucanases focused on genes from cyst and root knot nematodes and one migratory nematode, all belonging to the Tylenchoidea superfamily. In order to have a broader overview of the endoglucanase evolution in the infra-order Tylenchomorpha, the gene structure of six additional genes was incorporated in our study. These include the four *Radopholus similis *genes that were recently described [6] and two novel GHF5 endoglucanases from the migratory endoparasitic nematodes *Pratylenchus coffeae *and *Ditylenchus africanus*. The latter gene is the first GHF5 endoglucanase isolated from the superfamily Sphaerularioidea [31].

## Results and discussion

Glycosyl hydrolase family 5 (GHF5) endo-1,4-beta-glucanase genes have been identified in various plant-parasitic nematodes (PPN). These genes have different structures: all have a signal peptide and a catalytic domain, some have an additional linker and carbohydrate-binding module (CBM, type 2 or CBM2) and others only have a linker but no CBM2. This modular domain structure could have arisen by exon or domain shuffling, a process by which a chimeric protein is created by domains or segments of two different genes through recombination in intron regions. In bacteria, all endo-1,4-beta-glucanases are supposed to originate from a few progenitor sequences by mutation and domain shuffling [10] and Quillet *et al. *has proven the validity of this hypothesis for an endo-1,4-beta-glucanase of the bacterium *Myxococcus xanthus *[14]. The authors suggest that the catalytic domain and the CBM of this endoglucanase were acquired by independent horizontal transfers from different soil bacteria, and that these domains were assembled by exon shuffling to form a new gene.

While the ancient origin of the PPN GHF5 endoglucanases (horizontal gene transfer or not [12]) still is a matter of debate, in this study no support for an early exon/domain shuffling event after the independent gain of a catalytic domain (e.g. through evolution from an eukaryotic ancestor) and a CBM2 (e.g. through HGT from bacteria) in an ancestral PPN species could be found in the currently available sequence data.

### Endoglucanase genes of *Ditylenchus africanus *and *Pratylenchus coffeae*

Endo-1,4-beta-glucanase fragments were amplified by a degenerate PCR on genomic DNA of *D. africanus *and *P. coffeae*. This resulted in a fragment of 605 bp for *D. africanus *and a fragment of 443 bp for *P. coffeae*. Sequence analysis confirmed them to be endoglucanase fragments and the genes were named *Da-eng1 *and *Pc-eng1 *respectively. Full length sequences of the genes were obtained by genome walking. The length of these sequences from start till stop codon is 2007 bp for *Da-eng1 *and 1527 bp for *Pc-eng1*. The corresponding coding sequences were amplified from a cDNA pool and have respective lengths of 1395 bp and 1377 bp. *Da-eng1 *contains 5 introns whereas *Pc-eng1 *only has 3.

The putative endoglucanase proteins Da-ENG1 and Pc-ENG1 have an estimated molecular weight of 49 and 47 kDa respectively. Both have a predicted N-terminal signal peptide for secretion. A conserved domain search confirmed that the proteins are endoglucanases belonging to the glycosyl hydrolase family 5 (GHF5, pfam00150). In addition to the catalytic domain, both proteins have a linker and a carbohydrate-binding module (CBM) of family 2 (pfam00553).

### Phylogenetic analyses

In addition to the already known endoglucanase genes from PPN and the newly identified sequences in this study, we searched for the most homologous endoglucanase genes from other major lineages. Based on their sequence similarity to the PPN endoglucanases, one or more representative sequences were selected per lineage for further analyses. These included GHF5 endoglucanase sequences from the kingdom of Plantae, Animalia, Fungi, Protista and Bacteria (See additional file 1). Some of these genes consist of both a catalytic domain and a CBM, while others lack the CBM.

Separate alignments were made for the catalytic domain and the carbohydrate-binding module (CBM) of the genes. Since not all GHF5 endoglucanase genes include a CBM, the alignment and hence also the phylogenetic tree for the CBM contained less taxa. For both domains, alignment of the sequences was performed at both DNA and protein sequence level.

Alignments showed that plant sequences are not sufficiently similar to PPN GHF5 endoglucanases to be useful for phylogenetic analyses. Preliminary unrooted phylogenetic tree construction with the other endoglucanases revealed fungal sequences to be most divergent from PPN GHF5 endoglucanases. Therefore they were chosen as outgroup to construct rooted trees. All alignments were analyzed using Maximum Parsimony (MP), Maximum Likelihood (ML) and Bayesian methods.

No data from linker sequences have been included in this study (except for the presence of intron 22 in the linker of *Mi-eng1*, see further) because linkers show very little large-scale sequence homology. At DNA level, only linkers from very closely related genes show some degree of homology. This is not surprising as the same observation has been made for linkers from bacterial endoglucanases [10]. In general, nematode linkers, as well as bacterial linkers, are short and mainly composed of small or tiny amino acids like glycine, serine, threonine, alanine, proline, and lysine.

All evolutionary trees, whether based on DNA or protein data, showed similar clustering, independent of the method used.

**Figure 1:**
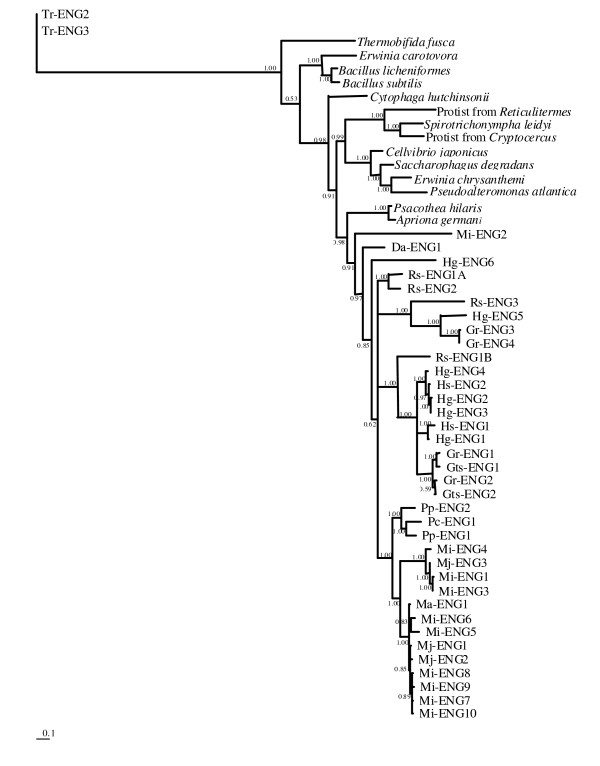
**Bayesian phylogenetic tree of the GHF5 catalytic domain**. Bayesian phylogenetic tree derived from a protein alignment of the catalytic domain of GHF5 endoglucanases from different prokaryotic and eukaryotic lineages, using the WAG model. The posterior probability is given on each node. The tree is rooted with the catalytic domain of two genes from the fungus *Trichoderma reesei*. The scale bar represents branch length (number of amino acid substitutions/site).

Figure 1 shows the Bayesian tree based on the protein alignment of the catalytic domain, using the fungal genes as outgroup. Protist genes form a well-confirmed monophyletic group nested in the paraphyletic bacterial cluster. Animal genes also form a monophyletic group in which beetle genes cluster separately from the PPN genes.

Within the PPN, Mi-ENG2, Da-ENG1 and Hg-ENG6 are at the base of the protein family but do not show a well-supported clustering. This grouping is correlated with their aberrant exon/intron structure (see further). The divergence of Da-ENG1 at sequence and structural level is not surprising since *Ditylenchus africanus *is the only species in our study belonging to the Sphaerularioidea and not to the Tylenchoidea.

A well-confirmed cluster groups the GHF5 endoglucanases of *Pratylenchus coffeae *and *P. penetrans *with those of the root knot nematodes (genus *Meloidogyne*). Cyst nematode (*Heterodera *and *Globodera*) endoglucanases are grouped together with some endoglucanases from *Radopholus similis *(classified in the Pratylenchidae family), Rs-ENG1B and Rs-ENG3. Although the positions of Rs-ENG1A and Rs-ENG2 are unresolved, these observations partially confirm recent phylogenetic trees derived from 28SrRNA data [32] revealing a closer relationship of *Radopholus similis *to Heteroderidae than to Pratylenchidae. This distinction was also observed on a morphological level by Luc [33], who found that the genus *Radopholus *can be distinguished from other genera in Pratylenchidae by its strong secondary sexual dimorphism and the distinctive lip pattern.

Paralogy between groups of proteins is well-confirmed by high posterior probabilities. For instance, Rs-ENG3 and Hg-ENG5 are paralogous to each other and to two orthologues from *Globodera rostochiensis *(Gr-ENG3 and Gr-ENG4). Also, Rs-ENG1B is paralogous to some intra-species or intra-genus duplicates from *Heterodera *and *Globodera *spp.

**Figure 2:**
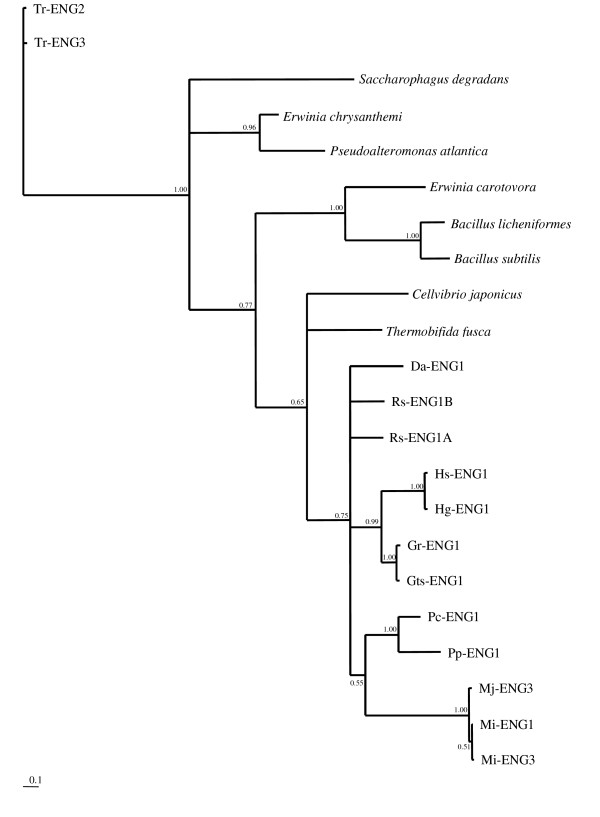
**Bayesian phylogenetic tree of the CBM of GHF5 endoglucanases**. Bayesian phylogenetic tree derived from a protein alignment of the carbohydrate-binding module (CBM) of GHF5 endoglucanases from different prokaryotic and eukaryotic lineages, using the WAG model. The posterior probability is given on each node. The tree is rooted with the CBM of two genes from the fungus *Trichoderma reesei*. The scale bar represents branch length (number of amino acid substitutions/site).

The Bayesian tree based on the CBM (Figure 2) reveals a similar clustering, and grouping of most PPN paralogues is again supported by maximum probability values (eg. Hs-ENG1 and Hg-ENG1; Pc-ENG1 and Pp-ENG1). However, posterior probabilities show low support for the relationships between genes from bacterial and PPN taxa. This lack of support for evolutionary relationships between nematode genes and genes from other major lineages precludes drawing conclusions about possible HGT events between bacterial and eukaryotic genomes.

Congruency between the obtained phylogenetic trees from the catalytic domain and the CBM was evaluated under the parsimony and likelihood criterion using the partition homogeneity and Shimodaira-Hasewaga (SH) tests. These analyses were run for all genes with a CBM as well as for a limited set excluding non-nematode taxa (PPN dataset). Interestingly, no statistical incongruence between all trees based on the GHF5 catalytic domain and on the CBM2 was found for the whole and the PPN dataset (Table 1). This observation suggests that these domains have evolved together.

**Table 1:** Congruence tests

	Partition homogeneity	SH-test
**All taxa**	0.24	0.16

**PPN**	0.76	0.11

Statistical significance values as obtained by applying the Partition homogeneity test and Shimodaira-Hasegawa (SH) test to test for congruence (P > 0.01: congruent) between the GHF5 catalytic domain and CBM2 evolutionary tracts from different eukaryotic and prokaryotic lineages (all taxa) and for a reduced dataset containing only genes from plant-parasitic nematodes (PPN).

**Figure 3:**
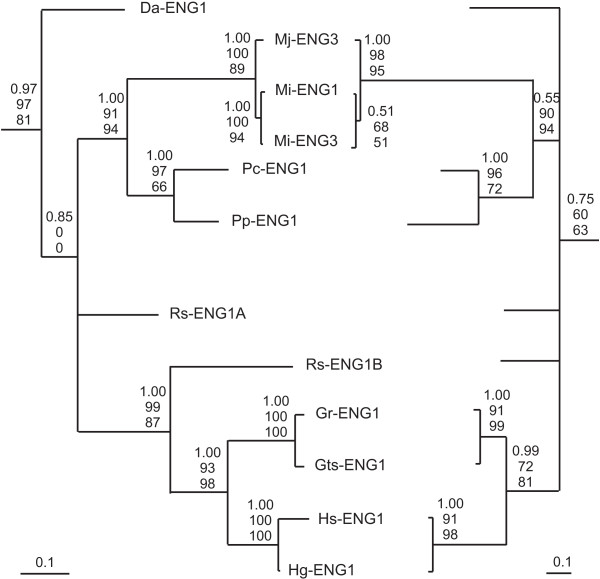
**Comparison between the Bayesian phylogenetic tree of the catalytic domain (left) and the tree of the carbohydrate-binding module (right) for all plant-parasitic nematode GHF5 endoglucanase genes with a CBM**. Scales of the corresponding trees are given in the lower corners. On each node, three values are given. From top to bottom: the posterior probabilities of the Bayesian analyses; the bootstrap values of the Maximum Likelihood analyses and the bootstrap values of the Maximum Parsimony analyses.

Figure 3 shows a comparison between the evolutionary trees of the GHF5 catalytic domain and the CBM domain for all PPN genes that contain both domains. Support values from Maximum Parsimony, Maximum Likelihood and Bayesian analysis are shown on the nodes. This representation demonstrates the possible congruent evolutionary tracts of both domains. Most clades are confirmed by both evolutionary trees with high posterior probabilities and/or bootstrap values.

### Investigation of gene/domain structure and intron properties

**Figure 4:**
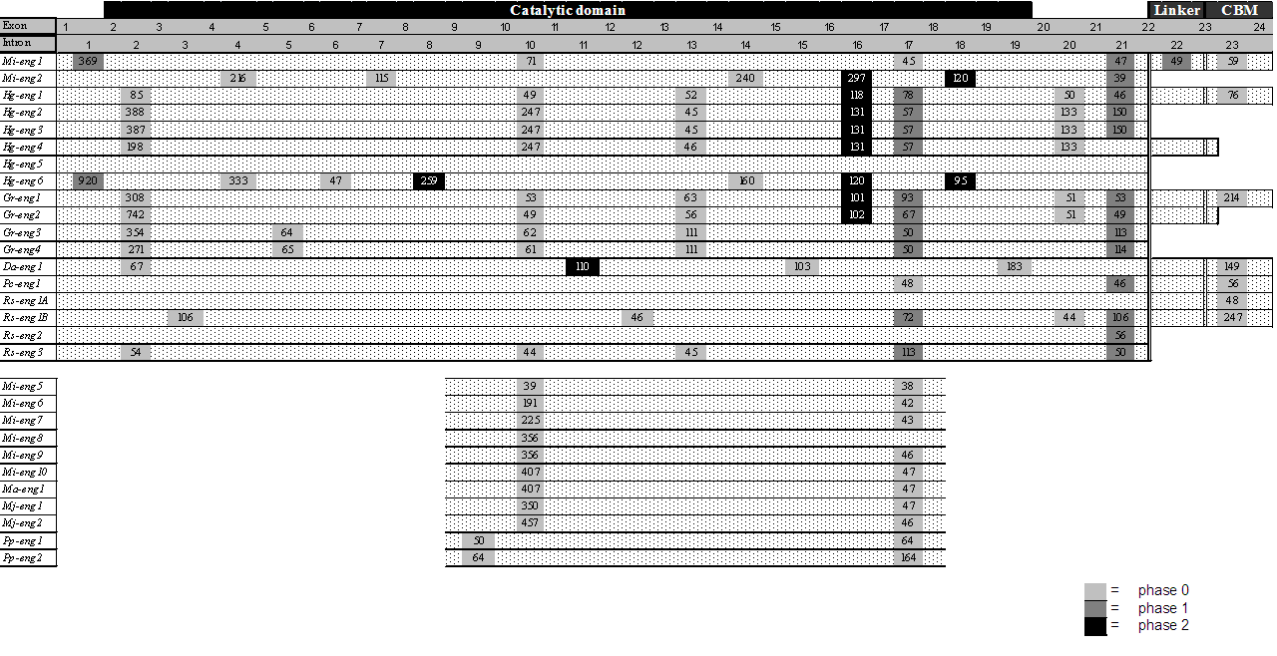
**Schematic overview of the exon/intron structure of all available genomic sequences from PPN GHF5 endoglucanase genes**. Introns are considered to be homologous when they appear at exactly the same location in the amino acid alignment. Phase of the introns is shown by gray-scale boxes. Length of the introns (in bp) is given inside each box. Domains, as identified by the SMART tool (PFAM database), are represented above the genes. Exon/intron and domain length are not drawn to scale.

The exon/intron gene structure of the PPN GHF5 endoglucanases was compared with the multiple protein sequence alignment. All introns and their properties are represented in Figure 4.

The ORF (Open Reading Frame) of PPN GHF5 endoglucanase genes is interrupted by maximum 8 introns (*Hg-eng1*) (Figure 4) with an average of 5.28 introns. The average length of each intron is 132 bp, making a total of 697 bp of intron per gene, which is smaller than the (already small) average intron size observed in the nematode lineage (467 bp per intron [34]).

The GC percentage per intron varies from 5% (intron 10 of *Mi-eng5*) to 58% (intron 2 of *Gr-eng1*), with an average for all introns (n = 110) of 31%. Apparently there are differences in GC percentage according to the species. For instance, the average GC% of all introns of *M. incognita *endoglucanase genes (n = 23) is 22%, whereas for *R. similis *introns (n = 13) it is 42%. This is probably related to the overall GC% of the coding sequences of a certain species. *M. incognita *coding sequences generally have a relatively low GC content (37%) [35], whereas *R. similis *coding sequences have a high GC content (54%) [36].

67% of the analysed introns are in phase 0, while only 17% correspond to phase 1 or phase 2. Phase 0 is highly over-represented in PPN endoglucanases, as also observed by Ledger *et al. *[13], with twice the expected value for an addition of intron with equal probability for the three codon sites. This is also considerably higher than the observed prevalence in *C. elegans *(47% [34]). 49% (54/110) of the exons are symmetrical. As a consequence of this high over-representation of phase 0 introns, symmetrical exons of type 0-0 (44%, 48/110) are largely in excess compared to symmetrical exons of type 1-1 (4%, 4/110) or type 2-2 (2%, 2/110). The observed excess of phase 0 introns can be due to the preferential retention of these introns by natural selection [37] or as a consequence of the burst of domain shuffling that has been shown to coincide with the big bang of metazoan radiation in the Cambrian period [15,38].

Exon-intron boundaries were compared for all introns observed in the available PPN GHF5 endoglucanase gene sequences. The great majority of the detected introns are of the GU-AG type. Only three intron positions (intron 13, 17a and 17b) show the alternative, but rare (0.6% in *C. elegans*) GC-AG splice site in a minority of the homologues. This non-classical splice site was already reported by Yan *et al. *[39].

**Figure 5:**
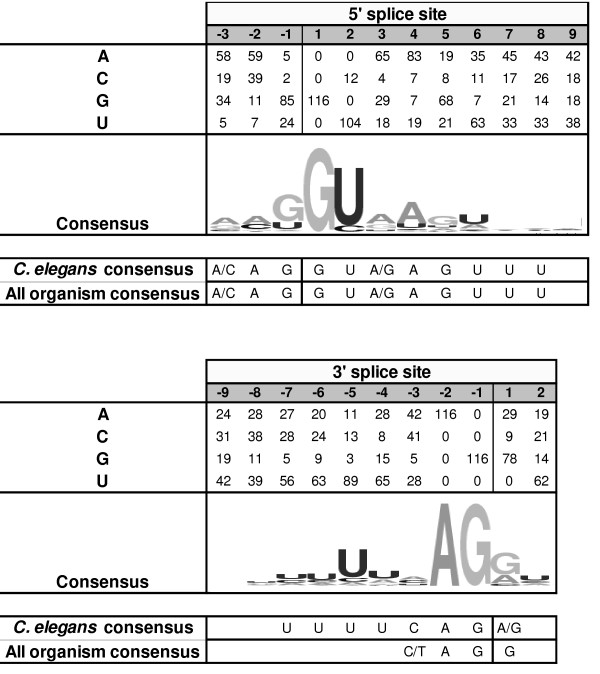
**Nucleotides at the 5' and 3' splice sites of all studied introns of PPN GHF5 endoglucanases**. Splicing occurs at the vertical lines. Positions are numbered with respect to the splice sites. A consensus splice site was derived and represented using Weblogo 3 [71]. For comparison, the consensus splice site of *C. elegans *and all organisms [40] is included below the deduced PPN consensus sequence.

A consensus 5' and 3' splice site was deduced from the exon-intron boundaries and is shown in Figure 5. Comparing this consensus with the previously described *C. elegans *splice site consensus [40] shows a low degree of conservation both at the 5' and 3' side. Low 5' splice site conservation has been shown to be correlated with a large intron number and the ability to generate alternatively spliced mRNAs [41].

Protosplice site tendency in PPN GHF5 endoglucanases is moderately high as 46% (11/24) of the introns show a consensus splice site that is completely consistent with the protosplice site (MAG/R), pointing to a probable recent origin of these introns.

Even though many clusters of closely spaced introns were detected in the aligned sequence, no evidence was found in our study, or in other studies, that proves their possible homology. Although intron sliding cannot be completely ruled out, the influence of this process is considered to be negligible (except for sliding by one base), and intron position diversity is indicated to be primarily arising by the gain or loss of introns during eukaryotic evolution [22]. Therefore, a pair of introns was required to occur in the exact same position in the aligned sequence of the orthologous/paralogous genes to be considered homologous.

Noteworthy, there is a strong conservation of the location and phase of introns within the PPN GHF5 gene family [13]. This conservation is evident among orthologous genes, but also among paralogues. Some intron positions are very well conserved (intron 2, 10, 13, 16, 17b, 21 and 23) while others only occur in one gene (intron 3, 6, 7, 8, 11, 12, 15, 19, 22).

Since spliceosomal introns diverge so rapidly that sequence similarity indicative of homology quickly vanishes, significant intron sequence conservation can only be expected in genes resulting from very recent duplications. Sequence comparisons between introns that are conserved in the majority of the dataset indeed showed similarities between paralogous genes. For instance, introns of *Gr-eng1 *and *Gr-eng2 *as well as *Gr-eng3 *and *Gr-eng4 *reveal remarkable similarity. This is also the case for introns in *Hg-eng2*, *Hg-eng3 *and *Hg-eng4*. The conserved introns of the *Meloidogyne *endoglucanase genes are also very similar, confirming the homology of these conserved introns and the paralogy of the genes.

As could be expected based on the low number of informative sites (14), Wagner Parsimony of the 1/0-data for intron presence/absence shows very low resolution (See additional file 2). Only some paralogous and orthologous genes with very similar exon/intron structure are grouped together with relatively high bootstrap support. For instance, *Mi-eng2 *and *Hg-eng6 *form a well-confirmed cluster. This is also the case for some groups of paralogues and orthologues from *H. glycines, G. rostochiensis *and *R. similis*. Generally, resolved branches show similar clustering as catalytic domain and CBM-based trees implying that intron presence data reflect the evolutionary tract of PPN GHF5 genes.

### Objections against an early domain shuffling event

With the currently available endoglucanase sequences, no evidence for the domain shuffling hypothesis could be found. No statistical incongruence between the evolutionary history of the GHF5 catalytic domain and the CBM2 in the whole dataset as well as in PPN only (Table 1 and Fig. 3) was detected. Some additional trends in the gene structure data also decline the domain shuffling idea.

Gilbert [42] proposed that correlation between intron locations and structural elements implies a role for introns in the evolution of proteins. The intronic architecture of the GHF5 endoglucanase genes was mapped onto a multiple protein sequence alignment in the context of the overall secondary structure of an archetypal GHF5 endoglucanase (See additional file 3), but there appears to be no general correlation.

However, some conserved intron positions are specifically located at the border of the catalytic domain and could therefore be implicated in domain shuffling. Introns 20 and 21 are found in the transition zone between the catalytic domain and the linker region in many genes. Intron 1 is located after the signal peptide, and before the start of the catalytic domain, but is only present in two genes. Successful domain shuffling requires that the domains are bordered by introns that are of the same phase, that is, that the domain is symmetrical [15], because shuffling of asymmetrical exons/domains will result in a frame-shift. The only possible symmetric, and hence movable, domain would be a 1-1 domain bordered by intron 1 and intron 21. Although 1-1 symmetrical domains are suggested to be frequently associated with domain shuffling events [38], the low conservation of intron 1 among the investigated gene structures (it is only present in 2 genes) as well as its well-conserved protosplice site point to a quite recent origin, and therefore make its involvement in a possible domain shuffling event very unlikely.

Finally, exon or domain shuffling is more likely to involve large introns as the frequency of recombination is known to be proportional to DNA length. Although we cannot exclude the possibility that larger introns were present in an ancestral GHF5 PPN endoglucanase, introns in the present-day nematode genes are extremely small (also for introns 20 and 21) and are therefore less likely to be involved in recombination.

### A model for the evolution of introns in the PPN GHF5 endoglucanases

**Figure 6:**
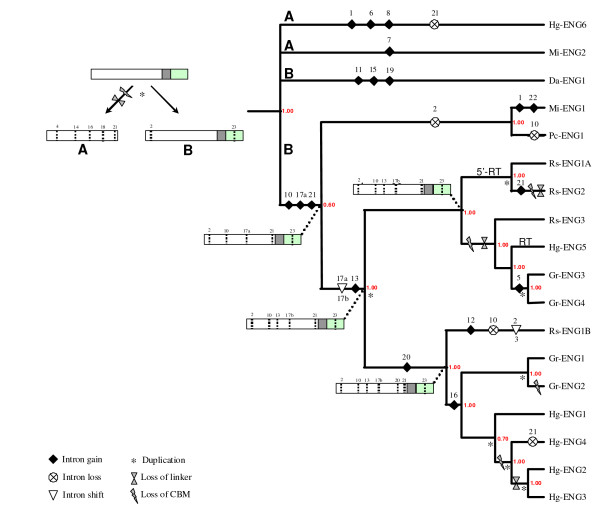
**Proposed model for GHF5 endoglucanase gene structure evolution in plant-parasitic nematodes**. The model was constructed by phylogenetic mapping of gene structure changes applying the parsimony principle (reducing the number of intron gains/losses during evolution), onto a Bayesian tree obtained from those PPN GHF5 genes for which gene structure information was available (Posterior probabilities are indicated in red for each node). Introns are numbered as specified in Figure 4. Assumed gene structures are schematically drawn for certain interesting nodes: the catalytic domain is represented by a white box, the linker is shown as a grey box and the CBM2 is shown as a green box; Intron presence is shown by dotted lines. RT: Reverse Transcription. See text for a more detailed explanation.

A model was constructed by combining the sequence-based evolutionary tracts with the intron data. Figure 6 shows a Bayesian tree obtained from the catalytic domain for all genes for which the exon/intron structure is available. The gene evolution model plotted onto this tree is based on the parsimony principle, meaning that the occurrence of intron gain and loss events during evolution is minimized.

The evolutionary tree based on the catalytic domains shows the genes with the same structure grouped in the same terminal branches, although the length and sequence of introns have diverged.

Our working hypothesis proposes that the divergence of the gene structure of the PPN GHF5 gene family is associated with the expansion of the number of members from an ancient or early eukaryotic ancestral gene. The possible congruent evolutionary tract of modules, as observed in this study (Table 1 and Fig. 3), suggests that the ancestral PPN GHF5 gene probably contained a CBM2. Bacterial endoglucanases show significant sequence homology in both domains and if the HGT hypothesis is true then the analyzed GHF5 endoglucanases likely evolved from a bacterial orthologue containing both domains. Present-day PPN GHF5 endoglucanase genes exhibit a large diversity of exon/intron structures, but protein sequences in the catalytic domain are relatively well-conserved. Generally, PPN GHF5 endoglucanases contain a rather high number of introns. It is known that paralogues undergo functional diversification, which tends to be accompanied by a weakened selection and the acceleration of sequence evolution [43]. Babenko *et al. *[44] observed two clear trends in the evolution of paralogous gene families: the evolution of their gene structure is notably dynamic, and this evolution involves more intron gains than losses. In addition, Logsdon *et al. *noticed that nematodes have a particularly high rate of intron turnover compared to other animals [45]. These tendencies are also recognizable in the PPN GHF5 gene family. Based on the recent indications that introns emerged during the earliest phases of eukaryotic evolution [30] our model starts from an intronless ancestral PPN GHF5 endoglucanase gene.

The exon/intron structure of *Hg-eng6 *and *Mi-eng2 *and their sequence divergence from the other genes indicate that they have probably originated from a different type of GHF5 gene (type A). This implies that the ancestral PPN GHF5 endoglucanase must have been duplicated already early in the Tylenchomorpha lineage. This early duplication event was also suggested by Ledger *et al. *[13]. During or shortly after this duplication one of the copies (type A) must have lost its linker and CBM2. All other genes are proposed to have evolved from the second copy (type B) containing linker and CBM2. Selection might have played a role in the preferential conservation of this type, possibly due to a positive effect of the CBM2. Whether intron 23 has been gained before, during or shortly after the duplication cannot be evaluated based on the available sequence data.

In the ancestral nematode of the lineage leading to the cyst nematodes and *Radopholus similis*, a second gene duplication took place. These nematodes all contain two divergent types of GHF5 endoglucanases within their genomes.

At intra-species level, the conserved gene structure of certain paralogues and the observation of some sequence conservation in their introns indicate a history of recent duplication events in the present-day genomes of plant-parasitic nematodes.

Ledger *et al. *[13] also proposed an evolutionary scheme for the evolution of GHF5 endoglucanases in plant-parasitic nematodes. Their model proposed two ancestral copies of the GHF5 endoglucanase gene: one with and one without CBM, which is in agreement with our data. However, the model of Ledger *et al. *[13] does not take duplication events in ancestral taxa (e.g. as we have detected in the ancestor of *Radopholus similis *and the cyst nematodes) into consideration and is therefore confusing in regard of gene and species evolution.

In some cases, intra-species duplicates lost their CBM and occasionally also their linker region. Generally, the observed variation in exon/intron structures between homologous and paralogous genes implies that introns have been gained and lost along all major lineages of the tree and at different time points during evolution. Concordant with the fact that the conservation of protosplice sites strongly supports the recent origin of an intron [46], recently gained introns in our model (intron 5, 6, 12, 15, 19 and 22) show a stronger conservation of the protosplice site (MAG|R) than older introns.

Our model implies some special evolutionary gene structure processes for which little information is available and that are therefore difficult to prove.

The phylogenetic hallmark of intron sliding is a distribution in which one intron is nested within the distribution of another [22]. We propose the occurrence of intron sliding for two introns in our model, namely sliding of intron 17a to intron 17b and intron 2 to intron 3, and this hypothesis is based on their close vicinity (1 nucleotide between 17a and 17b and 3 nucleotides between 2 and 3) and the apparent nested distribution. While the nested clustering theory of Stoltzfus *et al. *[22] pertains for these introns and they are located in very close vicinity, the fast evolution of intron sequences precludes the ability of finding significant sequence similarity at intron-level. In literature, the few established reports of intron sliding all involve very recent events [47-50]. Alternative mechanisms like separate gain and loss events might equally likely have been involved in the observed pattern of intron distribution.

The genes with no or only one intron probably have lost their introns by reverse transcription and subsequent homologous reinsertion of the synthesized cDNA, affecting either the whole gene or only a part of it. *Hg-eng5 *is an example of a gene where this process might have happened. We propose a similar mechanism for *Rs-eng1A *and *Rs-eng2*, involving a reverse-transcribed mRNA containing only a catalytic domain, but in this case the recombination must have been followed by a duplication event. The loss of introns through reverse transcription of mRNAs followed by the recombination of the synthesized cDNA in the genome [51-53] is hard to prove and an alternative gene duplication event cannot be ruled out because the genomes of plant-parasitic nematodes probably contain more GHF5 endoglucanase genes than currently available in databases. If a gene duplication would have given rise to these genes, then a closely related paralogue, generated in the same duplication event, should be found in the genome [54]. The impossibility to establish a clear relation between the intronless gene *Hg-eng5 *and any other gene from the same species suggests that reverse transcription followed by a homologous reinsertion is most probable. For *Rs-eng1A *and *Rs-eng2*, the reverse transcription event is supposed to have taken place earlier in evolution, involving only the 5' end, and should have been followed by a duplication event, the loss of linker and CBM and a gain of intron 21 in one of the paralogues.

## Conclusion

This study integrates previously known sequence information from mainly sedentary plant-parasitic nematodes and new data from different migratory nematode species. With the available data from PPN GHF5 endoglucanases and other major prokaryotic and eukaryotic lineages no evidence for an early domain shuffling event between the two domains (GHF5 catalytic domain and CBM2) present in these genes was found, suggesting parallel evolutionary tracts. Moreover, the phylogenetic data confirmed that some endoglucanases of the migratory species *Radopholus similis *are more related to genes from the Heteroderidae, while *Pratylenchus *spp. show a close relationship to the root knot nematodes.

Our evolutionary model for the PPN GHF5 gene family proposes an intronless ancestral GHF5 endoglucanase gene in plant-parasitic nematodes, consisting of a catalytic domain and a CBM2. Early in the evolution of plant-parasitic nematodes, the ancestral gene must have been duplicated, and numerous introns were gained. Later in evolution, some introns were lost, gained or shifted in certain lineages, and additional duplication events frequently took place. Our data reveal that loss of the CBM2 and in some cases also the linker region almost certainly occurred independently in different lineages.

While our data show that exon shuffling most probably did not lie at the origin of the emergence of the PPN GHF5 endoglucanase domain structure, more recent exon/domain shuffling events at lower taxonomic levels cannot be ruled out.

In this study the HGT hypothesis can neither be accepted nor rejected. Additional sequence data from other major eukaryotic lineages are awaited before a thorough and sound investigation of this problem can be realized. However, evidence was provided that it is very unlikely that an ancestral eukaryotic GHF5 catalytic domain was joined with a horizontally-acquired CBM2 in the early evolution of the PPN GHF5 domain structure. Therefore, HGT of the whole structural cassette (GHF5-linker-CBM2) remains one of the most likely explanations for its presence in both bacteria and PPN.

## Methods

### Nematode culture

*Pratylenchus coffeae *and *Ditylenchus africanus *were maintained on carrot disks in small petri dishes at a constant temperature of 25°C [55]. Nematodes were collected by rinsing the petri dishes with sterile demineralized water approximately 5 weeks (for *D. africanus*) or 8 weeks (for *P. coffeae*) after inoculation of the carrot disks. Collected nematodes of mixed stages were stored as a pellet at -20°C for DNA or RNA extraction.

### Cloning of endoglucanase genes

Genomic DNA was isolated from the nematodes according to Bolla *et al. *[56]. RNA extraction was done with TRIzol (Invitrogen) according to the manufacturer's instructions. First strand cDNA was synthesized using 1 μg RNA, 4 mM dNTP's, 0.5 μM oligodT primer (T_25_VN), 10 mM DTT, 50 mM Tris-HCl (pH 8.3), 75 mM KCl, 3 mM MgCl_2 _and 200 U Superscript II Reverse Transcriptase (Invitrogen). This reaction mixture was incubated for 2 h at 42°C.

A polymerase chain reaction (PCR) using degenerate primers ENG1 (TAYGTIATHGTIGAYTGGCA) and ENG2 (GTICCRTAYTCIGTIACRAA) [4] was performed on genomic DNA of *P. coffeae *and *D. africanus*. The reaction mixture contained 150 ng DNA, 0.5 μM of each primer, 4 mM each of dNTPs, 1.5 mM MgCl_2_, 20 mM Tris-HCl (pH8.3), 50 mM KCl and 1 U of *Taq *DNA polymerase (Invitrogen). The PCR conditions were as follows: 2' at 94°C followed by 35 cycles of 1' at 94°C, 1' at 52°C and 1' at 72°C. Resulting fragments were separated on a 0.5× TAE 1.5% agarose gel, excised and purified by the QIAquick Gel Extraction kit (Qiagen). Purified fragments were ligated into pGEM-T (Promega) and transformed in *E. coli *DH5α cells (Invitrogen). A colony PCR under standard conditions revealed the presence or absence of inserts. Plasmids of positive colonies were isolated using the Nucleobond AX kit (Machery-Nagel) and the insert was sequenced at the VIB Genetic Service Facility (VIB-GSF, Antwerp, Belgium).

To obtain the 5' and 3' genomic sequences of the endoglucanase fragments, the Genome Walker Universal kit (Clontech) was used according to the manufacturer's instructions. Primers used for the *P. coffeae *fragment were Pc-eng1-up1 (TTGGAGTCAATGGCCCGGATGG), Pc-eng1-up2 (GATGTTCGGATTGGAGCCATATTGC) to walk upstream en Pc-eng1-down1 (TCAAATCTCTGCTACACTCTCCAC) and Pc-eng1-down2 (CAAACAATCCCTCAGGGATAAGGC) to walk downstream. For the *D. africanus *fragment, Da-eng1-up1 (CCAGCGTATTTTTGTGCCATTTGG), Da-eng1-up2 (CTCTCAACCAATATCTTTGACTTAACAGC), Da-eng1-down1 (AATAGTCGCGTTTAGGACGTGGACGTC) and Da-eng1-down2 (TCCACTTCTACGCCGGCACTCAC) were used to walk up- and downstream respectively. The longest obtained fragments were cloned and sequenced as described above. Corresponding cDNA sequences of the endoglucanase genes were amplified by PCR using gene-specific primers Da-eng1-start (ATGAAATTCTTCGCCAGCCTCG) and Da-eng1-stop (TCAGCAATATCCATAAGACACAACGC) on the cDNA pool of *D. africanus *and Pc-eng1-start (ATGGCATTCACTTTGCTTTCC) and Pc-eng1-stop (TGTGAGCGCCACTGCCTGCTAA) on *P. coffeae *cDNA. These cDNA sequences were cloned and sequenced as described above. Obtained sequences were submitted to the GenBank database (*Da-eng1*: EU180235; *Pc-eng1*: EU176871).

Putative protein sequences were obtained by translating the cDNA sequences using the EMBOSS program "Transeq" [57]. The molecular weight of the proteins was estimated by the "pI/Mw" tool of the Expasy server http://us.expasy.org. Signal peptides were predicted using SignalP 3.0 [58].

### Sequence searches and alignments

Using BlastX, a GenBank search for all available endoglucanase gene sequences from nematode species was performed on May 21, 2007. For comparison, the most homologous endoglucanases from other eukaryotic and prokaryotic lineages were included in the dataset (See additional file 1).

Protein, DNA and mRNA sequences were downloaded. Domains were identified using the SMART tool [59], which is based on the PFAM database. Sequences were aligned using ClustalW [60] and then manually adjusted in BioEdit [61].

### Exon-intron structure analyses

The presence, location and phase of introns were evaluated for each open reading frame (ORF) by comparing DNA and mRNA sequences. Intron positions were mapped onto the sequence alignments. Intron phase was named 0 when the intron was located in-between two consecutive codons; 1 if an intron was located between the first and second codon nucleotides and 2 if an intron was found between the second and third codon nucleotides. The intron positions were mapped onto the secondary structure (See additional file 3). Positions of alpha helices and beta strands in the catalytic domain were deduced from an alignment of all nematode endoglucanases with an endoglucanase from *Erwinia chrysanthemi *(1EGZC), which has a known 3D structure. For the carbohydrate-binding module, the location of the beta strands was inferred by aligning the sequences to a CBM from an endoglucanase from *Cellulomonas fimi *(1EXG). GC percentages of the introns were calculated with the internet tool GC calculator http://www.genomicsplace.com/gc_calc.html.

### Phylogenetic analyses

Maximum Parsimony (MP) analyses were executed using PAUP* v4.0b10 [62], on the informative characters of the mRNA and protein alignments using the heuristic search option with random sequence addition (100 random replications) and TBR branch-swapping. Support for the different clusters was evaluated by bootstrap analysis (100 replicates).

Intron presence was coded by binary scoring (1/0) for presence/absence. Introns were only considered to be homologous when they appeared at exactly the same position and the same phase in the orthologous/paralogous genes. Although Dollo Parsimony is frequently used in intron evolution [26], recent evidence shows that this method tends to underestimate the number of introns in ancestral nodes and to overestimate the number of gains in branches leading to extant species [63] since it is based on the assumption that intron presence originated only once in the tree. Considering the fact that no consensus has been reached about the dominance of intron gain or loss during eukaryotic evolution, our 1/0-data were analysed using Wagner Parsimony [64].

Congruence between the obtained Maximum Parsimony trees based on catalytic domain and CBM was tested using the partition homogeneity test (HT_F_) as designated by Johnson & Soltis [65], which is analogous to the ILD (incongruence length difference) test of Farris *et al. *[66] as implemented in PAUP* v4.0b10 using the heuristic search option with random sequence addition (100 random replications) and TBR branch-swapping.

Hierarchical Likelihood Ratio Tests were performed on all datasets using Modeltest 3.5 [67] to determine the best-fitting evolutionary model. Estimated model parameters were applied in a Maximum Likelihood (ML) analysis in PAUP* v4.0b10 using the heuristic search option with random sequence addition, for DNA sequences. Tree-puzzle 5.2 [68] was used for ML analysis of protein sequences. Support for the different clusters was evaluated by bootstrap analysis (100 replicates).

In order to compare trees obtained from ML analyses, congruence between tree topologies derived from catalytic domain and CBM, was statistically evaluated using the likelihood-based test of Shimodaira & Hasewaga (SH-test) [69] as implemented in Tree-puzzle 5.2.

Bayesian analysis was run using MrBayes version 3.1.2 [70].

For the DNA datasets the best-fitting evolutionary model, as identified using Modeltest 3.5, was applied. For the protein datasets a mixed model of amino acid evolution was applied to allow model-jumping for fixed-rate models. Bayesian inference was run for 1,000,000 generations, and the first 25,000 generations were discarded as burn-in.

## Authors' contributions

TK did the phylogenetic analyses and drafted the manuscript. AH did the experimental work, i.e. cloning the genes, and was also involved in the phylogenetic analyses and the drafting of the manuscript. GG participated in the coordination of this study and critically revised the manuscript.

## Supplementary Material

Additional file 1 **List of available data**. Overview of all available endoglucanase gene sequences from nematode species and the most homologous endoglucanases from other eukaryotic and prokaryotic lineages that were included in our dataset. GenBank Accession Numbers and the type of available data are specified. Click here for file

Additional file 2 **Maximum Parsimony tree based on the intron data of GHF5 endoglucanases**. Consensus tree of 34 Maximum parsimonious trees based on intron presence/absence data of GHF5 endoglucanases from different prokaryotic and eukaryotic lineages. Wagner Parsimony was applied. The bootstrap value is given on each node. The tree is rooted with the CBM of two genes from the fungus *Trichoderma reesei*. Click here for file

Additional file 3 **Intron positions of a typical PPN GHF5 endoglucanase in relation to the secondary gene structure**. The position of alpha helices and beta strands was derived from comparison to known crystal structures of a GHF5 catalytic domain (1EGZC) and a CBM2 (1EXG). The signal peptide, catalytic domain, linker and CBM, are indicated in red, blue, yellow and green respectively. Transition zones are white. Arrows represent beta strands; boxes alpha helices. Introns are represented by a vertical black line and numbered in order of their occurrence, as specified in Figure 4. Click here for file
