# Supplementary figures and images for: Evolution of GHF5 endoglucanase gene structure in plant-parasitic nematodes: no evidence for an early domain shuffling event
Source: BMC Evol Biol. 2008 Nov 3;8:305. doi: 10.1186/1471-2148-8-305 (PMC2633302; doi:10.1186/1471-2148-8-305)

Additional file 2

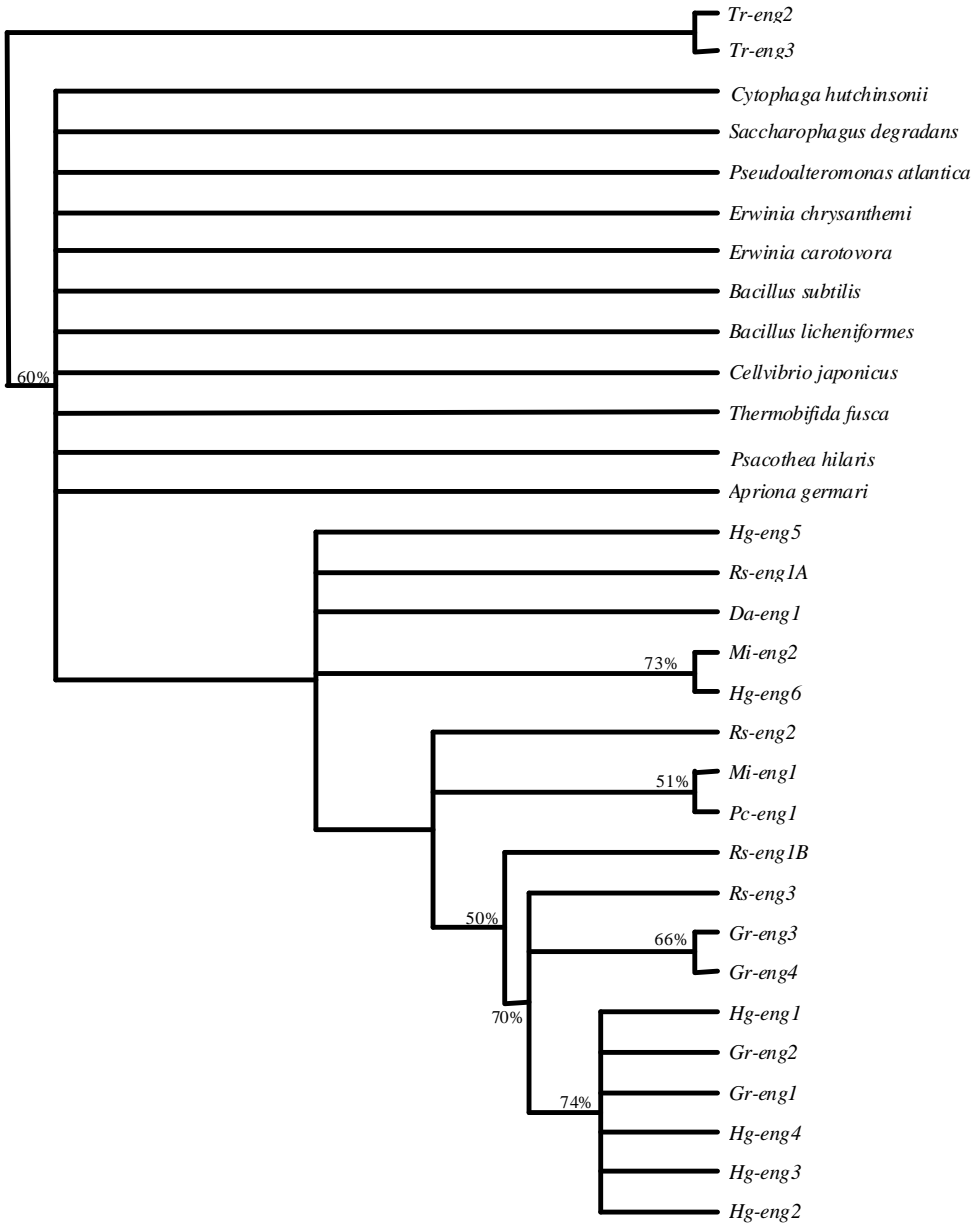

Supplement: Additional file 2 — Maximum Parsimony tree based on the intron data of GHF5 endoglucanases. Consensus tree of 34 Maximum parsimonious trees based on intron presence/absence data of GHF5 endoglucanases from different prokaryotic and eukaryotic lineages. Wagner Parsimony was applied. The bootstrap value is given on each node. The tree is rooted with the CBM of two genes from the fungus Trichoderma reesei. [file 1471-2148-8-305-S2.pdf]

**Additional file 3.**

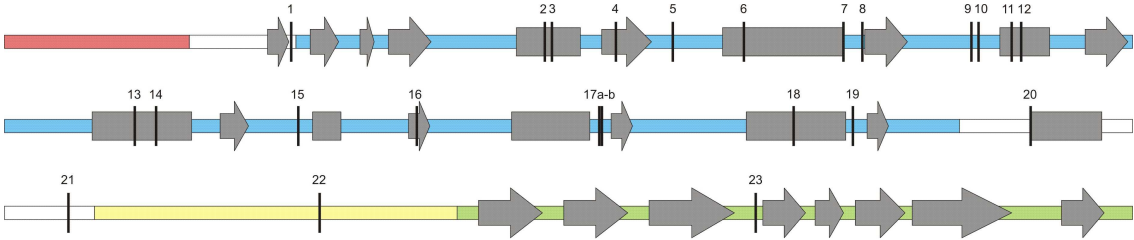

Supplement: Additional file 3 — Intron positions of a typical PPN GHF5 endoglucanase in relation to the secondary gene structure. The position of alpha helices and beta strands was derived from comparison to known crystal structures of a GHF5 catalytic domain (1EGZC) and a CBM2 (1EXG). The signal peptide, catalytic domain, linker and CBM, are indicated in red, blue, yellow and green respectively. Transition zones are white. Arrows represent beta strands; boxes alpha helices. Introns are represented by a vertical black line and numbered in order of their occurrence, as specified in Figure 4. [file 1471-2148-8-305-S3.pdf]
